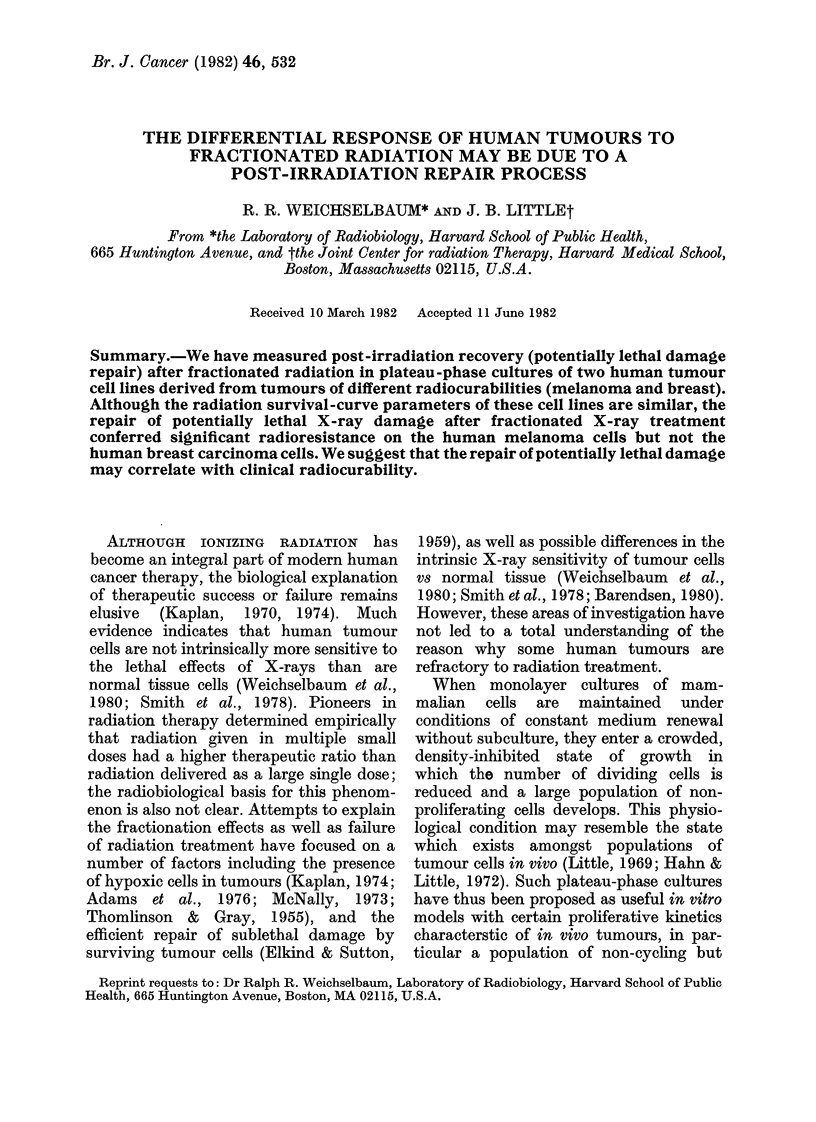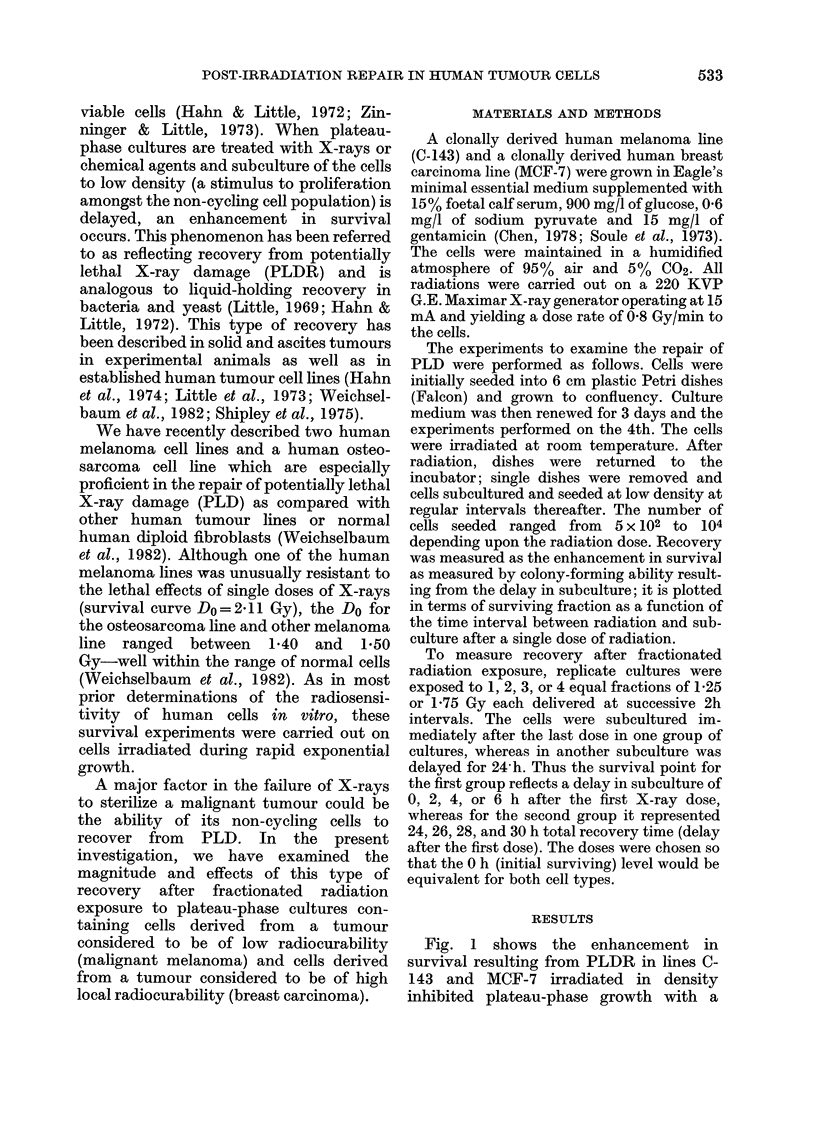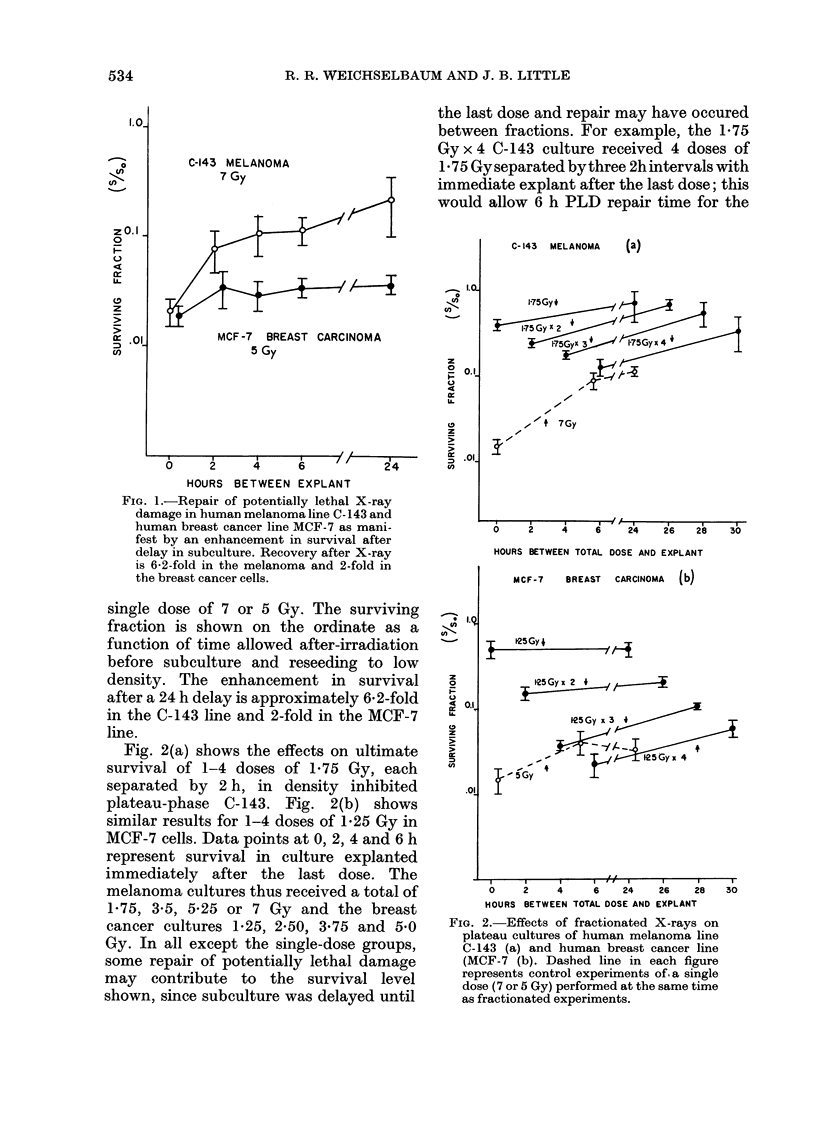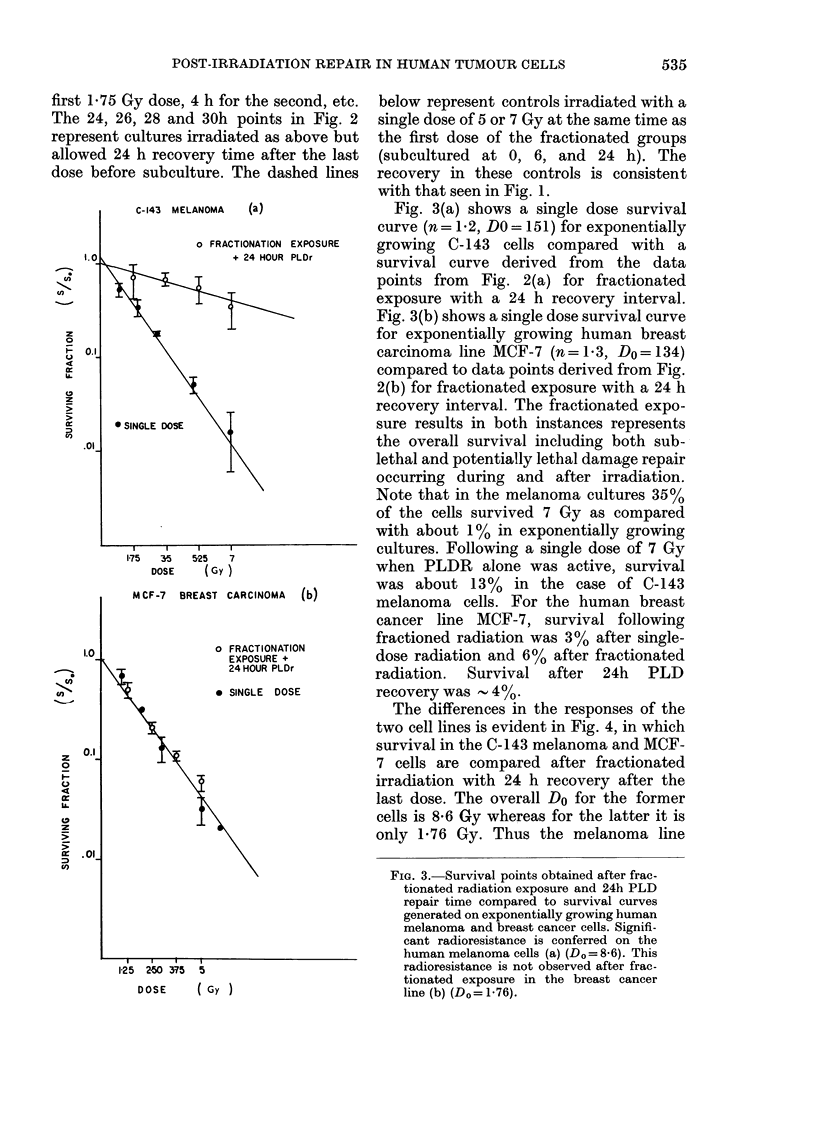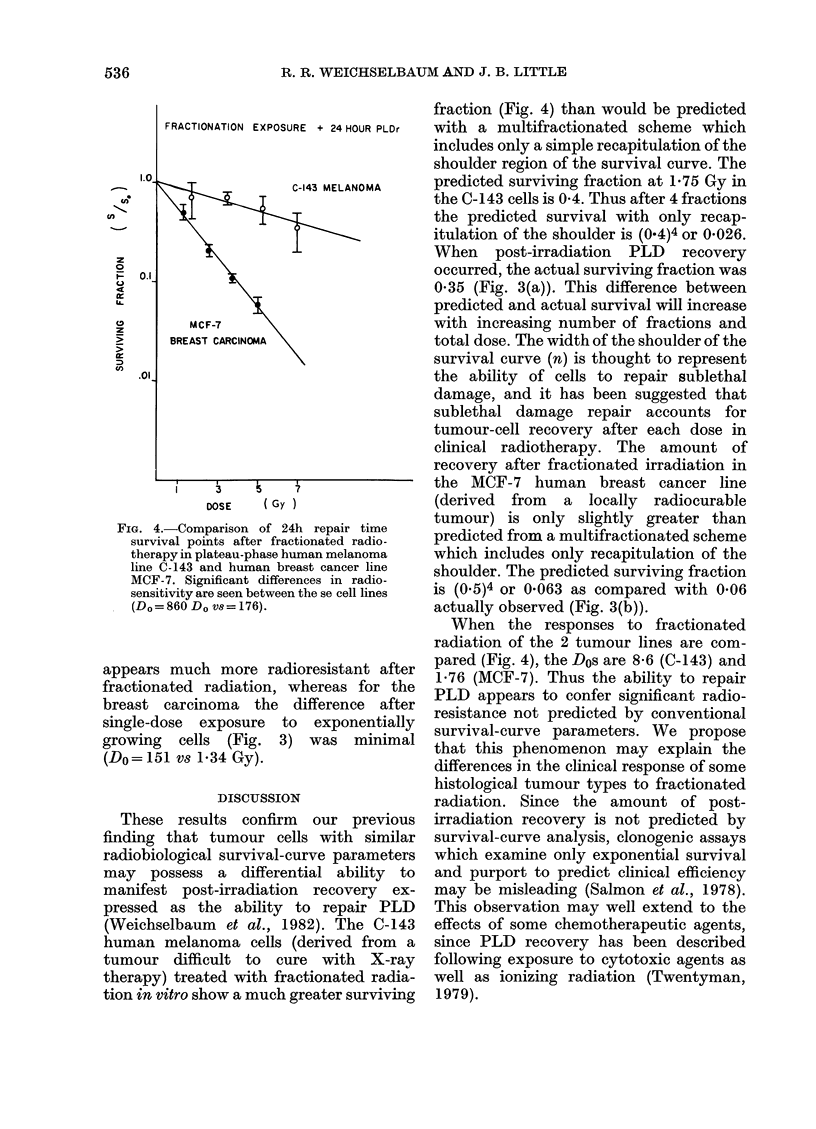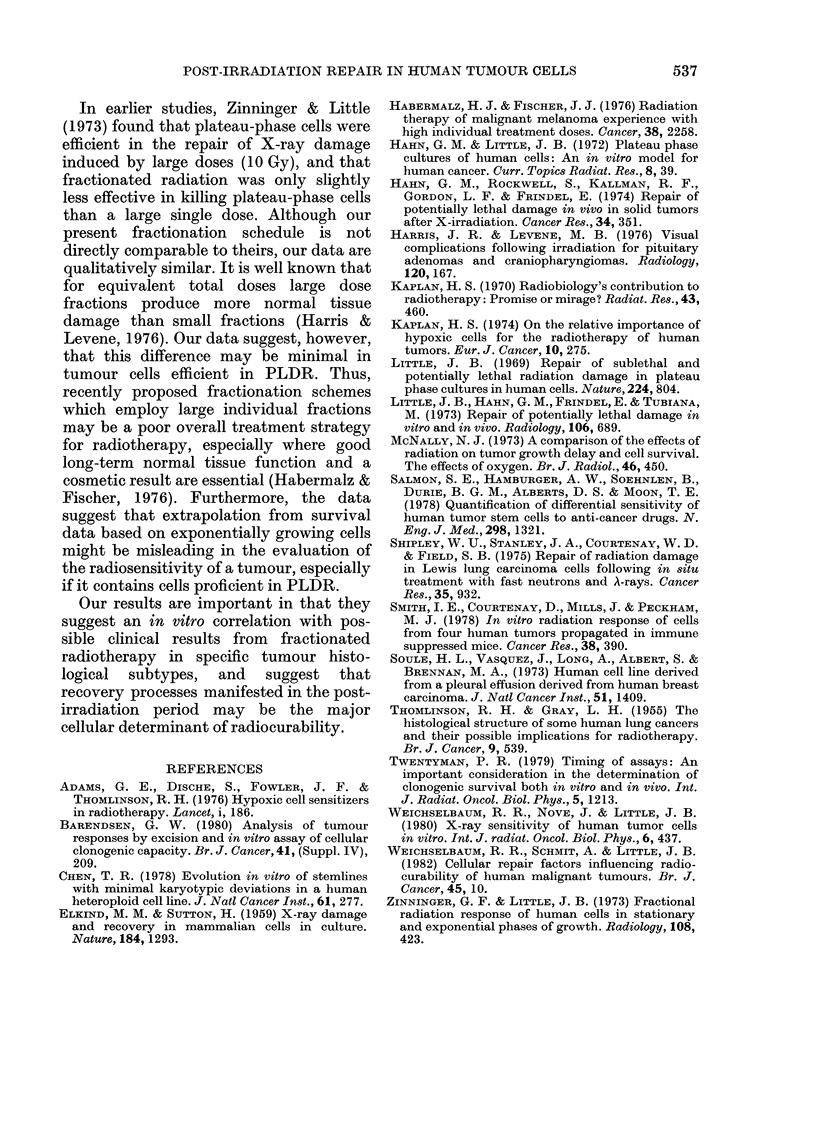# The differential response of human tumours to fractionated radiation may be due to a post-irradiation repair process.

**DOI:** 10.1038/bjc.1982.237

**Published:** 1982-10

**Authors:** R. R. Weichselbaum, J. B. Little

## Abstract

We have measured post-irradiation recovery (potentially lethal damage repair) after fractionated radiation in plateau-phase cultures of two human tumour cell lines derived from tumours of different radiocurabilities (melanoma and breast). Although the radiation survival-curve parameters of these cell lines are similar, the repair of potentially lethal X-ray damage after fractionated X-ray treatment conferred significant radioresistance on the human melanoma cells but not the human breast carcinoma cells. We suggest that the repair of potentially lethal damage may correlate with clinical radiocurability.


*Br. J. Cancer* (1982) **46**, 532

### THE DIFFERENTIAL RESPONSE OF HUMAN TUMOURS TO FRACTIONATED RADIATION MAY BE DUE TO A POST-IRRADIATION REPAIR PROCESS

R. R. WEICHSELBAUM* AND J. B. LITTLE†

*From *the Laboratory of Radiobiology, Harvard School of Public Health,* 665 *Huntington Avenue, and †the Joint Center for radiation Therapy, Harvard Medical School, Boston, Massachusetts* 02115, *U.S.A.*

Received 10 March 1982 Accepted 11 June 1982

**Summary.—We have measured post-irradiation recovery (potentially lethal damage repair) after fractionated radiation in plateau-phase cultures of two human tumour cell lines derived from tumours of different radiocurabilities (melanoma and breast). Although the radiation survival-curve parameters of these cell lines are similar, the repair of potentially lethal X-ray damage after fractionated X-ray treatment conferred significant radioresistance on the human melanoma cells but not the human breast carcinoma cells. We suggest that the repair of potentially lethal damage may correlate with clinical radiocurability.**

ALTHOUGH IONIZING RADIATION has become an integral part of modern human cancer therapy, the biological explanation of therapeutic success or failure remains elusive (Kaplan, 1970, 1974). Much evidence indicates that human tumour cells are not intrinsically more sensitive to the lethal effects of X-rays than are normal tissue cells (Weichselbaum *et al.*, 1980; Smith *et al.*, 1978). Pioneers in radiation therapy determined empirically that radiation given in multiple small doses had a higher therapeutic ratio than radiation delivered as a large single dose; the radiobiological basis for this phenomenon is also not clear. Attempts to explain the fractionation effects as well as failure of radiation treatment have focused on a number of factors including the presence of hypoxic cells in tumours (Kaplan, 1974; Adams *et al.*, 1976; McNally, 1973; Thomlinson & Gray, 1955), and the efficient repair of sublethal damage by surviving tumour cells (Elkind & Sutton, 1959), as well as possible differences in the intrinsic X-ray sensitivity of tumour cells *vs* normal tissue (Weichselbaum *et al.*, 1980; Smith *et al.*, 1978; Barendsen, 1980). However, these areas of investigation have not led to a total understanding of the reason why some human tumours are refractory to radiation treatment.

When monolayer cultures of mammalian cells are maintained under conditions of constant medium renewal without subculture, they enter a crowded, density-inhibited state of growth in which the number of dividing cells is reduced and a large population of non-proliferating cells develops. This physiological condition may resemble the state which exists amongst populations of tumour cells *in vivo* (Little, 1969; Hahn & Little, 1972). Such plateau-phase cultures have thus been proposed as useful *in vitro* models with certain proliferative kinetics characterstic of *in vivo* tumours, in particular a population of non-cycling but

Reprint requests to: Dr Ralph R. Weichselbaum, Laboratory of Radiobiology, Harvard School of Public Health, 665 Huntington Avenue, Boston, MA 02115, U.S.A.

viable cells (Hahn & Little, 1972; Zinninger & Little, 1973). When plateau-phase cultures are treated with X-rays or chemical agents and subculture of the cells to low density (a stimulus to proliferation amongst the non-cycling cell population) is delayed, an enhancement in survival occurs. This phenomenon has been referred to as reflecting recovery from potentially lethal X-ray damage (PLDR) and is analogous to liquid-holding recovery in bacteria and yeast (Little, 1969; Hahn & Little, 1972). This type of recovery has been described in solid and ascites tumours in experimental animals as well as in established human tumour cell lines (Hahn *et al.*, 1974; Little *et al.*, 1973; Weichselbaum *et al.*, 1982; Shipley *et al.*, 1975).

We have recently described two human melanoma cell lines and a human osteosarcoma cell line which are especially proficient in the repair of potentially lethal X-ray damage (PLD) as compared with other human tumour lines or normal human diploid fibroblasts (Weichselbaum *et al.*, 1982). Although one of the human melanoma lines was unusually resistant to the lethal effects of single doses of X-rays (survival curve $D_0 = 2{\cdot}11$ Gy), the $D_0$ for the osteosarcoma line and other melanoma line ranged between 1·40 and 1·50 Gy—well within the range of normal cells (Weichselbaum *et al.*, 1982). As in most prior determinations of the radiosensitivity of human cells *in vitro*, these survival experiments were carried out on cells irradiated during rapid exponential growth.

A major factor in the failure of X-rays to sterilize a malignant tumour could be the ability of its non-cycling cells to recover from PLD. In the present investigation, we have examined the magnitude and effects of this type of recovery after fractionated radiation exposure to plateau-phase cultures containing cells derived from a tumour considered to be of low radiocurability (malignant melanoma) and cells derived from a tumour considered to be of high local radiocurability (breast carcinoma).

### MATERIALS AND METHODS

A clonally derived human melanoma line (C-143) and a clonally derived human breast carcinoma line (MCF-7) were grown in Eagle's minimal essential medium supplemented with 15% foetal calf serum, 900 mg/l of glucose, 0·6 mg/l of sodium pyruvate and 15 mg/l of gentamicin (Chen, 1978; Soule *et al.*, 1973). The cells were maintained in a humidified atmosphere of 95% air and 5% $CO_2$. All radiations were carried out on a 220 KVP G.E. Maximar X-ray generator operating at 15 mA and yielding a dose rate of 0·8 Gy/min to the cells.

The experiments to examine the repair of PLD were performed as follows. Cells were initially seeded into 6 cm plastic Petri dishes (Falcon) and grown to confluency. Culture medium was then renewed for 3 days and the experiments performed on the 4th. The cells were irradiated at room temperature. After radiation, dishes were returned to the incubator; single dishes were removed and cells subcultured and seeded at low density at regular intervals thereafter. The number of cells seeded ranged from $5 \times 10^2$ to $10^4$ depending upon the radiation dose. Recovery was measured as the enhancement in survival as measured by colony-forming ability resulting from the delay in subculture; it is plotted in terms of surviving fraction as a function of the time interval between radiation and subculture after a single dose of radiation.

To measure recovery after fractionated radiation exposure, replicate cultures were exposed to 1, 2, 3, or 4 equal fractions of 1·25 or 1·75 Gy each delivered at successive 2h intervals. The cells were subcultured immediately after the last dose in one group of cultures, whereas in another subculture was delayed for 24 h. Thus the survival point for the first group reflects a delay in subculture of 0, 2, 4, or 6 h after the first X-ray dose, whereas for the second group it represented 24, 26, 28, and 30 h total recovery time (delay after the first dose). The doses were chosen so that the 0 h (initial surviving) level would be equivalent for both cell types.

### RESULTS

Fig. 1 shows the enhancement in survival resulting from PLDR in lines C-143 and MCF-7 irradiated in density inhibited plateau-phase growth with a

FIG. 1.—Repair of potentially lethal X-ray damage in human melanoma line C-143 and human breast cancer line MCF-7 as manifest by an enhancement in survival after delay in subculture. Recovery after X-ray is 6·2-fold in the melanoma and 2-fold in the breast cancer cells.

single dose of 7 or 5 Gy. The surviving fraction is shown on the ordinate as a function of time allowed after-irradiation before subculture and reseeding to low density. The enhancement in survival after a 24 h delay is approximately 6·2-fold in the C-143 line and 2-fold in the MCF-7 line.

Fig. 2(a) shows the effects on ultimate survival of 1–4 doses of 1·75 Gy, each separated by 2 h, in density inhibited plateau-phase C-143. Fig. 2(b) shows similar results for 1–4 doses of 1·25 Gy in MCF-7 cells. Data points at 0, 2, 4 and 6 h represent survival in culture explanted immediately after the last dose. The melanoma cultures thus received a total of 1·75, 3·5, 5·25 or 7 Gy and the breast cancer cultures 1·25, 2·50, 3·75 and 5·0 Gy. In all except the single-dose groups, some repair of potentially lethal damage may contribute to the survival level shown, since subculture was delayed until the last dose and repair may have occured between fractions. For example, the 1·75 Gy × 4 C-143 culture received 4 doses of 1·75 Gy separated by three 2h intervals with immediate explant after the last dose; this would allow 6 h PLD repair time for the

FIG. 2.—Effects of fractionated X-rays on plateau cultures of human melanoma line C-143 (a) and human breast cancer line (MCF-7 (b). Dashed line in each figure represents control experiments of a single dose (7 or 5 Gy) performed at the same time as fractionated experiments.

first 1·75 Gy dose, 4 h for the second, etc. The 24, 26, 28 and 30h points in Fig. 2 represent cultures irradiated as above but allowed 24 h recovery time after the last dose before subculture. The dashed lines below represent controls irradiated with a single dose of 5 or 7 Gy at the same time as the first dose of the fractionated groups (subcultured at 0, 6, and 24 h). The recovery in these controls is consistent with that seen in Fig. 1.

Fig. 3(a) shows a single dose survival curve ($n = 1{\cdot}2$, $D0 = 151$) for exponentially growing C-143 cells compared with a survival curve derived from the data points from Fig. 2(a) for fractionated exposure with a 24 h recovery interval. Fig. 3(b) shows a single dose survival curve for exponentially growing human breast carcinoma line MCF-7 ($n = 1{\cdot}3$, $D_0 = 134$) compared to data points derived from Fig. 2(b) for fractionated exposure with a 24 h recovery interval. The fractionated exposure results in both instances represents the overall survival including both sublethal and potentially lethal damage repair occurring during and after irradiation. Note that in the melanoma cultures 35% of the cells survived 7 Gy as compared with about 1% in exponentially growing cultures. Following a single dose of 7 Gy when PLDR alone was active, survival was about 13% in the case of C-143 melanoma cells. For the human breast cancer line MCF-7, survival following fractioned radiation was 3% after single-dose radiation and 6% after fractionated radiation. Survival after 24h PLD recovery was ~4%.

The differences in the responses of the two cell lines is evident in Fig. 4, in which survival in the C-143 melanoma and MCF-7 cells are compared after fractionated irradiation with 24 h recovery after the last dose. The overall $D_0$ for the former cells is 8·6 Gy whereas for the latter it is only 1·76 Gy. Thus the melanoma line

FIG. 3.—Survival points obtained after fractionated radiation exposure and 24h PLD repair time compared to survival curves generated on exponentially growing human melanoma and breast cancer cells. Significant radioresistance is conferred on the human melanoma cells (a) ($D_0 = 8{\cdot}6$). This radioresistance is not observed after fractionated exposure in the breast cancer line (b) ($D_0 = 1{\cdot}76$).

FIG. 4.—Comparison of 24h repair time survival points after fractionated radiotherapy in plateau-phase human melanoma line C-143 and human breast cancer line MCF-7. Significant differences in radiosensitivity are seen between the se cell lines ($D_0 = 860$ $D_0$ *vs* $= 176$).

appears much more radioresistant after fractionated radiation, whereas for the breast carcinoma the difference after single-dose exposure to exponentially growing cells (Fig. 3) was minimal ($D_0 = 151$ *vs* 1·34 Gy).

### DISCUSSION

These results confirm our previous finding that tumour cells with similar radiobiological survival-curve parameters may possess a differential ability to manifest post-irradiation recovery expressed as the ability to repair PLD (Weichselbaum *et al.*, 1982). The C-143 human melanoma cells (derived from a tumour difficult to cure with X-ray therapy) treated with fractionated radiation *in vitro* show a much greater surviving fraction (Fig. 4) than would be predicted with a multifractionated scheme which includes only a simple recapitulation of the shoulder region of the survival curve. The predicted surviving fraction at 1·75 Gy in the C-143 cells is 0·4. Thus after 4 fractions the predicted survival with only recapitulation of the shoulder is $(0{\cdot}4)^4$ or 0·026. When post-irradiation PLD recovery occurred, the actual surviving fraction was 0·35 (Fig. 3(a)). This difference between predicted and actual survival will increase with increasing number of fractions and total dose. The width of the shoulder of the survival curve ($n$) is thought to represent the ability of cells to repair sublethal damage, and it has been suggested that sublethal damage repair accounts for tumour-cell recovery after each dose in clinical radiotherapy. The amount of recovery after fractionated irradiation in the MCF-7 human breast cancer line (derived from a locally radiocurable tumour) is only slightly greater than predicted from a multifractionated scheme which includes only recapitulation of the shoulder. The predicted surviving fraction is $(0{\cdot}5)^4$ or 0·063 as compared with 0·06 actually observed (Fig. 3(b)).

When the responses to fractionated radiation of the 2 tumour lines are compared (Fig. 4), the $D_0$s are 8·6 (C-143) and 1·76 (MCF-7). Thus the ability to repair PLD appears to confer significant radioresistance not predicted by conventional survival-curve parameters. We propose that this phenomenon may explain the differences in the clinical response of some histological tumour types to fractionated radiation. Since the amount of post-irradiation recovery is not predicted by survival-curve analysis, clonogenic assays which examine only exponential survival and purport to predict clinical efficiency may be misleading (Salmon *et al.*, 1978). This observation may well extend to the effects of some chemotherapeutic agents, since PLD recovery has been described following exposure to cytotoxic agents as well as ionizing radiation (Twentyman, 1979).

In earlier studies, Zinninger & Little (1973) found that plateau-phase cells were efficient in the repair of X-ray damage induced by large doses (10 Gy), and that fractionated radiation was only slightly less effective in killing plateau-phase cells than a large single dose. Although our present fractionation schedule is not directly comparable to theirs, our data are qualitatively similar. It is well known that for equivalent total doses large dose fractions produce more normal tissue damage than small fractions (Harris & Levene, 1976). Our data suggest, however, that this difference may be minimal in tumour cells efficient in PLDR. Thus, recently proposed fractionation schemes which employ large individual fractions may be a poor overall treatment strategy for radiotherapy, especially where good long-term normal tissue function and a cosmetic result are essential (Habermalz & Fischer, 1976). Furthermore, the data suggest that extrapolation from survival data based on exponentially growing cells might be misleading in the evaluation of the radiosensitivity of a tumour, especially if it contains cells proficient in PLDR.

Our results are important in that they suggest an *in vitro* correlation with possible clinical results from fractionated radiotherapy in specific tumour histological subtypes, and suggest that recovery processes manifested in the post-irradiation period may be the major cellular determinant of radiocurability.

### REFERENCES

ADAMS, G. E., DISCHE, S., FOWLER, J. F. & THOMLINSON, R. H. (1976) Hypoxic cell sensitizers in radiotherapy. *Lancet*, i, 186.

BARENDSEN, G. W. (1980) Analysis of tumour responses by excision and *in vitro* assay of cellular clonogenic capacity. *Br. J. Cancer*, **41**, (Suppl. IV), 209.

CHEN, T. R. (1978) Evolution *in vitro* of stemlines with minimal karyotypic deviations in a human heteroploid cell line. *J. Natl Cancer Inst.*, **61**, 277.

ELKIND, M. M. & SUTTON, H. (1959) X-ray damage and recovery in mammalian cells in culture. *Nature*, **184**, 1293.

HABERMALZ, H. J. & FISCHER, J. J. (1976) Radiation therapy of malignant melanoma experience with high individual treatment doses. *Cancer*, **38**, 2258.

HAHN, G. M. & LITTLE, J. B. (1972) Plateau phase cultures of human cells: An *in vitro* model for human cancer. *Curr. Topics Radiat. Res.*, **8**, 39.

HAHN, G. M., ROCKWELL, S., KALLMAN, R. F., GORDON, L. F. & FRINDEL, E. (1974) Repair of potentially lethal damage *in vivo* in solid tumors after X-irradiation. *Cancer Res.*, **34**, 351.

HARRIS, J. R. & LEVENE, M. B. (1976) Visual complications following irradiation for pituitary adenomas and craniopharyngiomas. *Radiology*, **120**, 167.

KAPLAN, H. S. (1970) Radiobiology's contribution to radiotherapy: Promise or mirage? *Radiat. Res.*, **43**, 460.

KAPLAN, H. S. (1974) On the relative importance of hypoxic cells for the radiotherapy of human tumors. *Eur. J. Cancer*, **10**, 275.

LITTLE, J. B. (1969) Repair of sublethal and potentially lethal radiation damage in plateau phase cultures in human cells. *Nature*, **224**, 804.

LITTLE, J. B., HAHN, G. M., FRINDEL, E. & TUBIANA, M. (1973) Repair of potentially lethal damage *in vitro* and *in vivo*. *Radiology*, **106**, 689.

MCNALLY, N. J. (1973) A comparison of the effects of radiation on tumor growth delay and cell survival. The effects of oxygen. *Br. J. Radiol.*, **46**, 450.

SALMON, S. E., HAMBURGER, A. W., SOEHNLEN, B., DURIE, B. G. M., ALBERTS, D. S. & MOON, T. E. (1978) Quantification of differential sensitivity of human tumor stem cells to anti-cancer drugs. *N. Eng. J. Med.*, **298**, 1321.

SHIPLEY, W. U., STANLEY, J. A., COURTENAY, W. D. & FIELD, S. B. (1975) Repair of radiation damage in Lewis lung carcinoma cells following *in situ* treatment with fast neutrons and λ-rays. *Cancer Res.*, **35**, 932.

SMITH, I. E., COURTENAY, D., MILLS, J. & PECKHAM, M. J. (1978) *In vitro* radiation response of cells from four human tumors propagated in immune suppressed mice. *Cancer Res.*, **38**, 390.

SOULE, H. L., VASQUEZ, J., LONG, A., ALBERT, S. & BRENNAN, M. A., (1973) Human cell line derived from a pleural effusion derived from human breast carcinoma. *J. Natl Cancer Inst.*, **51**, 1409.

THOMLINSON, R. H. & GRAY, L. H. (1955) The histological structure of some human lung cancers and their possible implications for radiotherapy. *Br. J. Cancer*, **9**, 539.

TWENTYMAN, P. R. (1979) Timing of assays: An important consideration in the determination of clonogenic survival both *in vitro* and *in vivo*. *Int. J. Radiat. Oncol. Biol. Phys.*, **5**, 1213.

WEICHSELBAUM, R. R., NOVE, J. & LITTLE, J. B. (1980) X-ray sensitivity of human tumor cells *in vitro*. *Int. J. radiat. Oncol. Biol. Phys.*, **6**, 437.

WEICHSELBAUM, R. R., SCHMIT, A. & LITTLE, J. B. (1982) Cellular repair factors influencing radiocurability of human malignant tumours. *Br. J. Cancer*, **45**, 10.

ZINNINGER, G. F. & LITTLE, J. B. (1973) Fractional radiation response of human cells in stationary and exponential phases of growth. *Radiology*, **108**, 423.